# Effect of CRISPR/Cas9-Edited PD-1/PD-L1 on Tumor Immunity and Immunotherapy

**DOI:** 10.3389/fimmu.2022.848327

**Published:** 2022-03-01

**Authors:** Yanxin Xu, Chen Chen, Yaxin Guo, Shengyun Hu, Zhenqiang Sun

**Affiliations:** ^1^ Department of Colorectal Surgery, The First Affiliated Hospital of Zhengzhou University, Zhengzhou, China; ^2^ School of Life Sciences, Zhengzhou University, Zhengzhou, China; ^3^ School of Basic Medical Sciences, Zhengzhou University, Zhengzhou, China

**Keywords:** CRISPR/Cas9, PD-1, PD-L1, tumor immunity, immunotherapy

## Abstract

Clustered regularly interspaced short palindromic repeats/CRISPR-associated nuclease9 (CRISPR/Cas9) gene editing technology implements precise programming of the human genome through RNA guidance. At present, it has been widely used in the construction of animal tumor models, the study of drug resistance regulation mechanisms, epigenetic control and innovation in cancer treatment. Tumor immunotherapy restores the normal antitumor immune response by restarting and maintaining the tumor-immune cycle. CRISPR/Cas9 technology has occupied a central position in further optimizing anti-programmed cell death 1(PD-1) tumor immunotherapy. In this review, we summarize the recent progress in exploring the regulatory mechanism of tumor immune PD-1 and programmed death ligand 1(PD-L1) based on CRISPR/Cas9 technology and its clinical application in different cancer types. In addition, CRISPR genome-wide screening identifies new drug targets and biomarkers to identify potentially sensitive populations for anti-PD-1/PD-L1 therapy and maximize antitumor effects. Finally, the strong potential and challenges of CRISPR/Cas9 for future clinical applications are discussed.

## Introduction

Cancer is a genetic disease accompanied by the accumulation of a variety of mutations (1). Tumor immunotherapy has become one of the most promising therapeutic strategies in tumor treatment compared with the high recurrence rate of metastasis, drug resistance and poor prognosis of traditional therapies (2). Tumor cells actively evade immune detection by activating associated negative regulatory pathways (also known as checkpoints), thus inhibiting the immune response of the human body. Among the numerous checkpoints, cytotoxic T lymphocyte protein 4 (CTLA4) and programmed cell death protein 1 (PD-1) are by far received much attention. CTLA4 controls T cell activation by competing with the costimulatory molecule CD28 to bind to the shared ligands CD80 and CD86 (3). The binding of PD-1 on the surface of T cells to Programmed death ligand 1(PD-L1) on tumor cells is a major obstacle in the cancer immune cycle, inducing T cell apoptosis and inhibiting the activation and proliferation of T cells (4). Fortunately, genetic modification of tumor immunity can be achieved through the CRISPR/Cas9 system. The CRISPR/Cas9 system, derived from the acquired immune defense mechanism of bacteria, enables accurate editing of specific regions of the genome by simple and rapid Watson Crick base pairing between sgRNA and target genes (5, 6). Currently, the CRISPR/Cas9 system has derived many variants such as nickase Cas9 (nCas9), nuclease-deactivated Cas9 (dCas9) and so on, which have been widely used in a variety of cell types and organisms to achieve diversity and ease of use (7, 8). In this review, we summarize CRISPR/Cas9 technology centering on gene editing, gene screening and clinical application of tumor immunity PD-1/PD-L1, as well as its strong potential and existing problems in the field of future tumor therapy.

## CRISPR/Cas9 Technology

**Figure 1:**
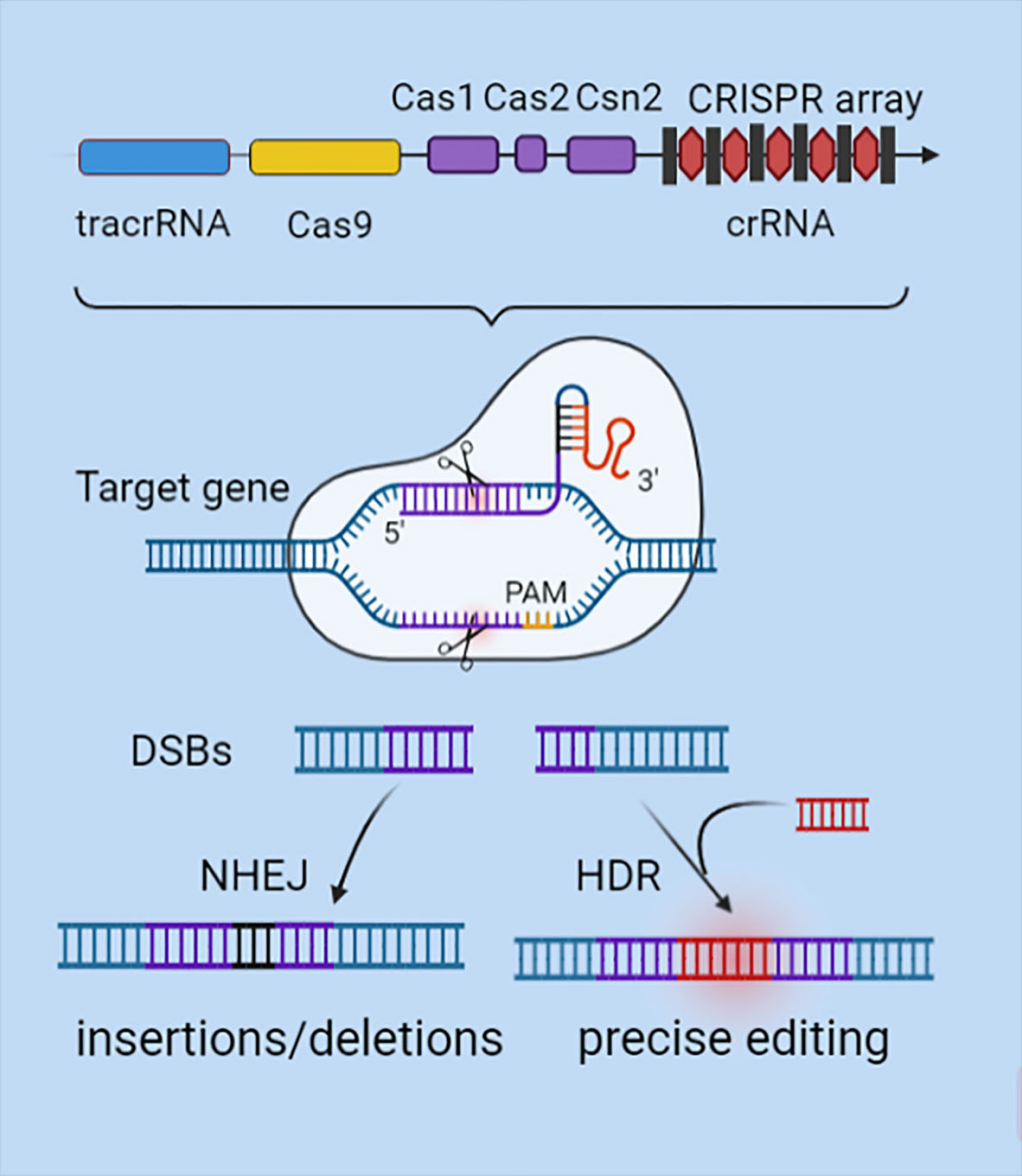
CRISPR/Cas9 gene editing mechanism. CRISPR’s sequence consists of a leader, repeats, and spacers. SgRNA is formed by the combination of specific crRNA and tracrRNA that functions as a scaffold. SgRNA can recruit the Cas9 to anchor to the target gene by using RNA-DNA base pairing at the 5'-terminal specific binding sequence. After verifying the PAM motif, Cas9 plays a shearing role to generate DSBs. DSBs are activated by NHEJ or HDR mechanisms to repair mutations, knock in or knock out specific genes, and modify genes.

Tumors are characterized by highly heterogeneous molecular characteristics and carry a wide range of gene mutations (9). This may be the main cause of tumor drug resistance and recurrence (10). Gene editing technology is in urgent need of decoding the genetic code at a deeper level and implementing precision medicine. Genome editing can modify the human genome and change biogenesis, which has the potential of targeted therapy to cure diseases (11). The CRISPR/Cas9 system is a gene editing technology guided by the principle of RNA-DNA complementary pairing (12). CRISPR/Cas9 technology is used to repair mutations, knock in or knock out specific genes, and modify genes to explore the mechanisms of tumorigenesis, development, and metastasis for precision therapy. The CRISPR sequence is responsible for the production of crRNA and tracrRNA (**Figure 1**). The two can be synthesized into a single-stranded guide RNA (sgRNA) (6, 13), which guides Cas9 nuclease to bind to the complementary target gene and cut near the protospacer adjacent motif (PAM) region to introduce DNA double strand breaks(DSBs) (5, 14). At DSBs, Non-homologous end-joining (NHEJ) repair pathway is activated to randomly insert or delete several bases, and efficiently produce INDEL mutations (15). Alternatively, Homologous directed repair (HDR) can occur under a DNA repair template with high homology (16). Compared with Zinc finger endonuclease (ZFN) (17) and Transcription activator-like effector nuclease (TALEN) (18) technologies, the CRISPR/Cas9 system abandons the traditional chimeric nuclease protein domain-DNA recognition design concept and avoids the multiple fusion process in multitarget gene editing (19–21). Therefore, it has the advantages of high specificity, strong operability and convenient systematic analysis, making it the preferred tool for gene editing in eukaryotes. CRISPR/Cas9 gene editing technology has been widely used in the construction of animal tumor models, the study of drug resistance regulation mechanisms, epigenetic control and tumor immunotherapy (22–24). In particular, CRISPR/Cas9 also shows great potential in exploring tumor immune mechanisms, biomarker screening, and clinical trials and therapies.

## Tumor Immunity

The occurrence and development of tumours are closely related to the infiltration of immune cells, immune modification and immune escape in the tumour microenvironment (1, 25, 26). Tumour immunity promotes tumour progression by changing tumour biological characteristics (27), screening tumour cells adapted to the microenvironment for survival (28) or establishing a suitable tumour microenvironment (29), and so on. Among them, immune checkpoints such as PD-1 and CTLA4 play an important role. Under normal physiological conditions, the binding of PD-1 and PD-L1 can down-regulate the activity of T cells and prevent additional damage of cytotoxic effector molecule and autoimmunity, so it is called immune checkpoint (30) A Study confirmed that tumor cells often express negative costimulatory signals such as PD-L1, leading to the failure activation of T cell activation. At the same time, tumor-infiltrating lymphocytes (TILs) usually express elevated levels of PD-1 due to chronic stimulation of tumor antigens (31). PD-1/PD-L1 blockade enhances T cell response through multiple underlying mechanisms, altering the outlook for cancer treatment. For example, PD-1/PD-L1 blockade may depend on proliferation of "precursors of exhausted" T (TPEX) cells rather than reversal of T cell depletion procedures (32). It has been found that PD-L1 blockade in conjunction with Alarmin HMGN1 peptide seems to activate anti-tumor TPEX cells and promote their amplification, but does not deplete T cells (33). In addition, PD-1 posttranslational modifications such as glycosylation maintain its stability and membrane expression, and mediate immunosuppressive function. Antibodies targeting glycosylated PD-1 may recognize the heavy glycan moieties of PD-1 and have a higher affinity (34).

## CRISPR/Cas9 in Tumor Immunity

Tumor immunity is regulated by many factors and ways. For example, iron poisoning can induce immunogenic apoptosis or necroptosis in cancer cells, which activates antitumor immunity (35). Autophagy regulation inhibits the response through selective degradation of MHC class I molecules, reduces the activity of the STING1 pathway, and promotes tumor immune escape (36). In recent years, CRISPR/Cas9 technology has received increasing attention in tumor immune regulation. CRISPR/Cas9 plays a role in the IFN signaling pathway, ERK/MAPK signaling pathway, Wnt/β-catenin signaling pathway, PI3K/AKT signaling pathway and other mechanisms in chronic inflammation and tumor immune resistance. For example, Wang et al. found that knockout of SHP2 using CRISPR/Cas9 gene editing inhibited SHP2 activity and enhanced tumor-intrinsic IFNγ signaling, resulting in increased chemoattractive cytokine release and cytotoxic T cell recruitment, as well as increased tumor cell surface Increased expression of MHC class I and PD-L1. In addition, SHP2 inhibition reduced the differentiation and suppressive function of immunosuppressive myeloid cells in the tumor microenvironment (37). The study by Yang et al. found that successful nuclear localization of CRISPR/Cas9 ensured efficient destruction of PD-L1 and PTPN2. Inhibition of PTPN2 can alleviate the inhibition of the JAK/STAT pathway and promote tumor susceptibility to CD8+ T cells dependent on IFN-γ, thereby further amplifying the adaptive immune response (38). Therefore, the combination of CRISPR/Cas9 technology with tumor immunotherapy has great potential for development. Immune checkpoint block is one of the most valuable individualized tumor treatment options. For example, the anti-PD-1/PD-L1 antibodies, nivolumab, pembrolizumab, and atezolizumab have shown significant advantages in melanoma, non-small-cell lung cancer (NSCLC), and urothelial carcinoma, and have been approved by the Food and Drug Administration (39–41). However, there remains inflammatory side effects (42, 43), and the overall survival rate is not significantly improved. In addition, CPISPR/Cas9 technology provides a multifunctional and convenient operation mode for the transformation of engineered T cells (44, 45). Genetically engineered T cells have the ability to kill tumor cells as well as significant therapeutic effects. Gene editing technology enables precise genetic modification of T cells, further improving chimeric antigen receptor-engineered T cell immunotherapy. This solves the problem of mass production of therapeutic immune cells and enables the genetic engineering of multiple T cells to meet the clinical needs for the treatment of complex types of cancer.

## CRISPR/Cas9-Edited PD-1/PD-L1 in the Tumor Immune Evasion

**Figure 2:**
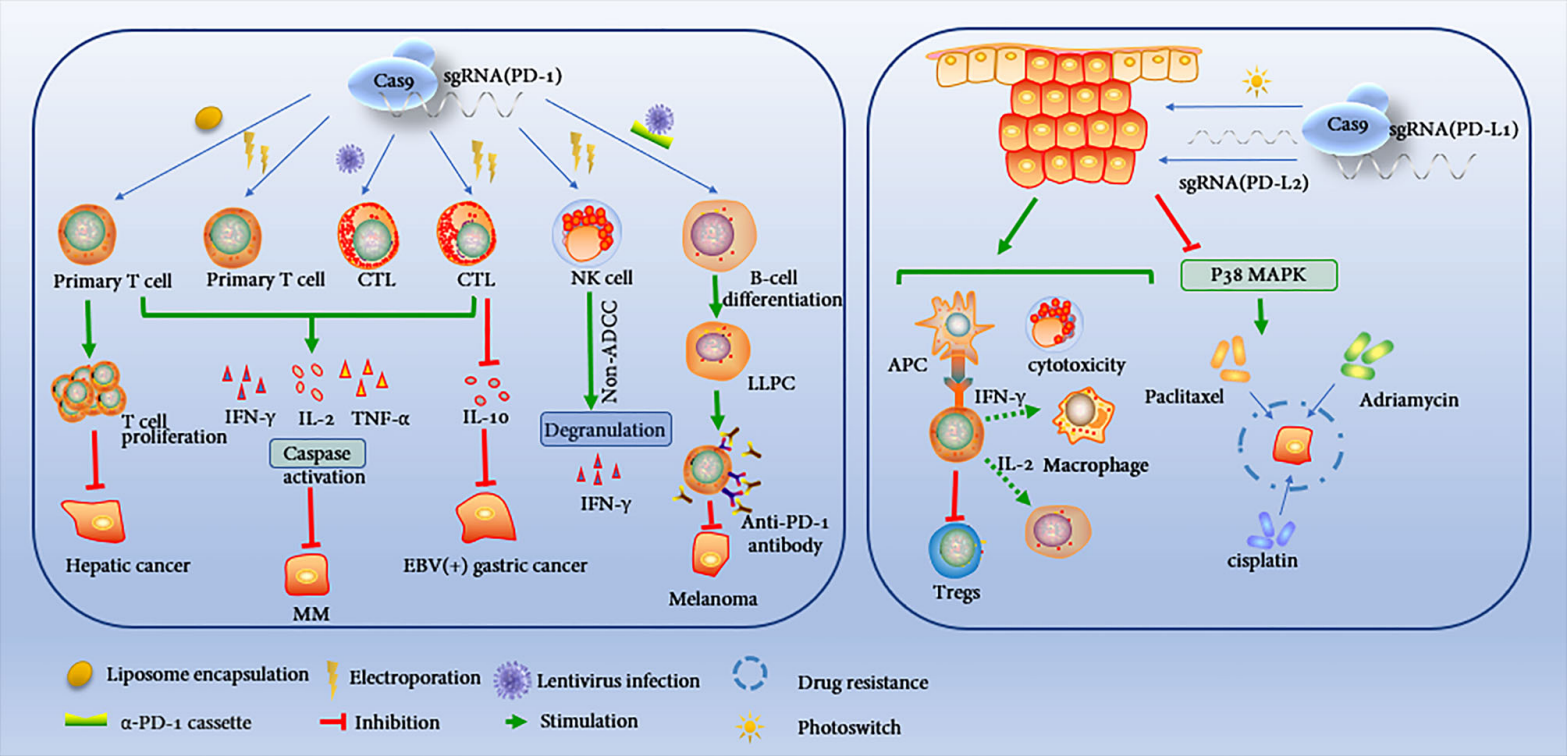
CRISPR/Cas9 directly edits PD-1/PD-L1 to regulate tumor immunity. CRISPR/Cas9 technology can edit the PD-1 gene in primary T cells, CTLs, NK cells and B cells through different delivery methods to enhance antitumor immunity through different mechanisms. CRISPR/Cas9 knockdown of PD-L1 in tumor cells promotes tumor antigen presentation, immune cell proliferation and cytotoxicity in the tumor microenvironment, and improves tumor chemotherapy resistance.

PD-1/PD-L1 has been identified as a negative immunomodulatory molecule that promotes immune evasion of tumor cells. Most patients treated with commercially targeted PD-1/PD-L1 antibodies have achieved a higher cure rate, prolonged survival and fewer recurrence and metastasis events (46–48). However, there are still some patients who are insensitive to targeted PD-1/PD-L1 antibodies or suffer side effects or drug-related inflammatory adverse events (49). The CRISPR/Cas9 technique maximizes PD-1/PD-L1 deletion at the genomic level, thereby saving the function of lymphocytes in the tumour microenvironment. However, whether it corrects the inactivation of targeted antibodies remains unclear (**Figure 2**).

### CRISPR/Cas9 Engineered Immune Cells

In primary T cells, researchers electroperforated plasmids encoding sgRNA and Cas9 to knock out PD-1, which upregulated IFN-γ production and enhanced cytotoxicity without affecting the viability of primary T cells (50). Similarly, Lu specifically knocked out the PD-1 gene in primary T cells using CRISPR/Cas9 genome editing tools coated with liposomes. This greatly stimulated T cell activation by dendritic cells (DCs) and demonstrated enhanced anticancer potential both *in vivo* and *in vitro* (51). Some studies have confirmed that CRISPR/Cas9 is an effective system to knock out PD-1 in cytotoxic T lymphocytes (CTLs). On the one hand, PD-1 KO CTLs can reduce the number of Tregs or inhibit Treg activity and recruit more effector cells. On the other hand, it regulates the secretion of cytokines and activates caspase, thereby inhibiting tumor growth *in vivo* and *in vitro* and prolonging survival (52–54). Cas9/sgRNA specifically integrates the α-PD-1 box into the GAPDH site of B lymphocytes. Surprisingly, the edited B lymphocytes differentiated into typical long-lived plasma cells (LLPCs) both *in vitro* and *in vivo*. These cells continuously secrete new antibodies that inhibit human melanoma growth in xenograft tumor mice by antibody-mediated checkpoint blocking (55). Cas9 has also improved natural killer cell-based cancer immunotherapy. PD-1-deficient NK cells increased lethality through non-antibody-dependent cell-mediated cytotoxicity (ADCC) pathways and enhanced degranulation and cytokine production (56). In summary, CRISPR/Cas9 provides a powerful and effective protocol for editing PD-1 genes in a variety of immune cells to block immune checkpoints.

### CRISPR/Cas9 Engineered Tumor Cells

PD-1/PD-L1 maintains high expression on the surface of various malignant tumors, which is associated with poor prognosis of the disease. PD-L1 leads to T cell dysfunction and tumor evasion from immune surveillance by binding to T cell suppressor receptors (57). After CRISPR/Cas9 reduced the expression level of PD-L1 on tumor cells, CD4 T cells, CD8 T cells, NK cells and CD11c M1 macrophages increased significantly in the tumor microenvironment, while regulatory T cells decreased. The mRNA levels of IFN-γ, TNF-α, interleukin (IL) -2, IL-12A, CXCL9 and CXCL10 were significantly increased, while the mRNA levels of IL-10, vascular endothelial growth factor, CXCL1 and CXCL2 were significantly decreased (58). Based on CRISPR/Cas9, scientists developed a photoactivated photoswitching system constructed from mg polyethylene imine derivative and plasmid PX330/SGPD-L1. The system effectively genetically destroys the PD-L1 gene in large cancer cells and even cancer stem cell-like cells (59). A vectorless multichannel gene editing system was designed in which multiple Cas9 RNPs inhibit both PD-L1 and programmed death ligand 2(PD-L2) ligands. It can significantly increase Th1 cytokine production of cytotoxic CD8 T cells, resulting in a synergistic cytotoxic effect (60). PD-L1 not only mediates tumor immune escape but also makes cancer cells resistant to chemotherapy. CRISPR/Cas9 modification of the tumor cell surface antigen PD-L1 can reduce chemotherapy resistance or produce synergistic effects in combination with chemoradiotherapy (61). For instance, the survival time of mice with the ovarian cancer in the PD-L1-KO group was significantly longer than that in the control group, and the therapeutic effect was enhanced when combined with cisplatin (58). Wu et al. found that CRISPR/cas9-mediated knockout of B7-H1 sensitized cancer cells to chemotherapy and targeting B7-H1 with a monoclonal antibody also sensitized cancer cells to chemotherapy. Furthermore, B7-H1 increases ERK activation in melanoma cells and maintains p38 MAPK activation in triple-negative breast cancer cells, and ERK activation plays a key role in cell survival and drug resistance (62). Targeting PD-L1 in osteosarcoma also increased drug sensitivity to adriamycin and paclitaxel. Doxorubicin and paclitaxel are commonly used to treat osteosarcoma. However, many patients with osteosarcoma are resistant to doxorubicin and paclitaxel chemotherapy. Liao et al. used the MTT assay to evaluate the role of PD-L1 in the resistance of osteosarcoma cells to doxorubicin and paclitaxel. They found that the results of the MTT assay indicated that PD-L1 may be involved in the drug resistance of osteosarcoma and may become a clinical potential target for therapy (63). In general, CRISPR/Cas9 technology not only improves the tumor immune microenvironment by knocking out PD-L1, but also breaks through tumor chemotherapy resistance.

## Indirect Regulation of PD-1/PD-L1 by CRISPR/Cas9

The occurrence and development of tumors is a complex pathological process involving the interaction of multiple regulatory molecules and their downstream signaling pathways. CRISPR/Cas9 has enabled further understanding of the regulatory mechanism of immune checkpoint inhibitors (ICIs) and promising biomarkers to help patients stratify and coordinate targeted anti-PD-1/PD-L1 therapy.

### Regulation of Antigen Presentation

Upregulation of antigen presenting-related genes of mouse MHC class I molecules, peptide transporters, peptide cleavages and transporter-MHC interactions was observed in lung squamous cell carcinoma lines with CRISPR deletion of WEE1 (64). This suggests the possibility of combining ICI with DNA damage induction therapy to treat laryngeal squamous cell carcinomas (LSCC). In addition, CRISPR-targeted EZH2 knockout reduced histone H3K27me3 modification on the B2 M promoter and restored antigen presentation. Anti-PD-1 antibodies also bypass the resistance of tumor cells to T cell-mediated killing (65). Therefore, the combination of EZH2-targeting with anti-PD-1 therapy may increase treatment susceptibility in head and neck squamous cell carcinoma (HNSCC). CRISPR/Cas9 promoted T cell infiltration in the tumor microenvironment by editing Atrx, and increased tumor cell antigen presentation after IFN-γ stimulation, thus enhancing the ICI response in NSCLC (66). Antigen presentation is a key step in initiating adaptive immunity, and CRISPR/Cas9 enhances antigen presentation to enhance antitumor immunity.

### Regulation of Tumor-Associated Macrophages (TAM)

A study found that CRISPR/Cas9 knockdown of Cxcr2 reduced the level of PD-L1 by downregulating C-MYC, thereby accelerating the change in the phenotype of M1 macrophages and suppressing immune escape. Therefore, Cxcr2 knockout is a potentially effective method to block PD-L1 (67). It has also been found that immunocompetent mice with CRISPR/Cas9 knockdown of VEGFC are more likely to develop invasive tumors than immunodeficient mice. These tumors showed downregulation of activated lymphocytes and upregulation of M2 macrophage markers and PD-L1 (68). Thus, targeted therapy for VEGFC must be considered comprehensively. In addition, Li used CRISPR/Cas9 to develop histone chaperone Asf1a deficiency, which coordinated with anti-PD-1 immunotherapy by promoting macrophage M1 polarization and T cell activation (69). CRISPR/Cas9 downregulates the expression of PD-L1 and reverses the tumor-promoting state of TAMs by regulating upstream molecules, thus playing an antitumor role.

### Regulating Signal Pathways

With the binding of PD-1 to PD-L1, tyrosine phosphatase is recruited to the cytoplasmic inhibitory motifs of PD-1 and dephosphorylates TCR, thereby inhibiting the downstream IFN, PI3K-AKT and Wnt/β-catenin signaling pathways to promote tumor progression (70, 71). CRISPR/Cas9 synergistically targets PD-1/PD-L1 therapy by regulating downstream signaling pathways. THE interferon (IFN) signaling pathway is a major component of natural immunity, and tumor cells may play a direct defensive role against IFN-mediated cytotoxicity by upregulating PD-L1. Knockout of SHP2 by CRISPR/Cas9 in cancer cells enhanced the expression of MHC class I and PD-L1 proteins in cancer cells through IFN-γ signaling and enhanced the response against PD-1 blockade in cogene mouse models (37). Programmable unlockable nanoencapsulation CRISPR technology (PUN) has hierarchical response characteristics. PUN mitigates inhibition of the JAK/STAT pathway by knocking out the protein tyrosine phosphatase PTPN2 in tumor cells, which relies on IFN-γ to promote antigen presentation and growth inhibition, further amplifying adaptive immunity to PD-L1 blockade (38). The PI3K-AKT signaling pathway is mainly involved in regulating the glycolysis process of immune cells (72). Studies found that CRISPR/Cas9-based GLO1 deletion changed glucose metabolism and redox homeostasis by modulating TXNIP, and PD-L1 expression was reduced at both the gene and protein levels (73). The Wnt//β-catenin pathway, which is extremely active in a variety of cancers, may also influence tumor immunity in addition to driving tumorgenesis and metastasis (74, 75). Researchers constructed an aptamer/peptide-functionalized genome-editing system, which significantly knocked out β-catenin and downregulated the Wnt//β-catenin pathway (76). After knockout, the expression of tumor immunosuppressor-related proteins (PD-L1 and CD47) and proliferation-related proteins (C-MYC and Cyclin D1) was significantly downregulated, which also reversed the immunosuppression induced by PD-L1 in the presence of IFN-γ. These findings provide a strong incentive for CRISPR/Cas9 to explore IFN, PI3K-Akt, and Wnt//β-catenin signaling pathways and strategies to enhance tumor immune invasion, as a means to increase tumor sensitivity to immune checkpoint blockade therapy.

## CRISPR/Cas9 Screening in PD-1/PD-L1 Immunotherapy

The occurrence and progression of tumors are closely related to gene mutations (1). Genome-wide screening can comprehensively and fairly grasp the changes in genes in cells, reveal the genetic basis behind specific phenotypes and assist in the development of new therapies. Advances in whole-genome screening in mammalian systems have been hampered by time, size constraints and inefficient double-allelic mutations in mouse culture (77). In recent years, CRISPR/Cas9 technology has provided genome-scale gRNA libraries in the presence of gRNA-guided Cas9 endonucases to generate mutant cell libraries, which serve as a perfect platform for phenotypic screening (78). At present, Cas9 technology has been used in the laboratory, and various sgRNA libraries constructed have revealed the internal molecular regulatory mechanisms of tumor proliferation and metastasis (79), tumor drug resistance (80, 81), tumor immune escape (82) and other malignant behaviors. Below, we summarize the recent progress of CRISPR/Cas9 technology in screening PD-1/PD-L1 and potential regulatory molecules in immune-related cells and tumor cells.

CRISPR technology was used to conduct genome-wide screening of tumor-infiltrating CD8 T cells *in vivo*. They reidentified typical immunotherapy targets such as PD-1 and TIM-3, as well as other novel sites, and verified the region of CRISPR in vivo screening of CD8 T cells for tumour infiltration (83). Another study identified Fut8, a gene involved in the core focusing pathway, as a positive regulator of PD-1 expression on the cell surface through genome-wide functional loss screening of the CRISPR-Cas9 system. Inhibition of Fut8 can reduce PD-1 expression on the cell surface, enhance T cell activation, and eradicate tumors more effectively (84). Using genome-wide CRISPR and metabolic inhibitor screening, Wang determined that nicotinamide phosphoribotransferase (NAMPT) is required for T cell activation and demonstrated that NAD+ supplementation significantly enhances anti-PD-1 immunotherapy in a mouse solid tumor model (85). This demonstrates the feasibility of CRISPR/Cas9 screening technology in discovering PD-1-related regulatory factors and provides a more comprehensive strategy for in-depth exploration of the PD-1 regulatory mechanism.

In addition to screening in T cells, CRISPR/Cas9 also showed great strength in the study of PD-L1 expressed in tumor cells. At the transcriptional level, CRISPR/Cas9 screening confirmed that ALK activated STAT3 and eventually induced PD-L1 expression through the effects of the transcription factors IRF4 and BATF3 on PD-L1 gene enhancer regions (86). At the translation level, the overexpression of translation initiation factor eIF5B in lung adenocarcinoma allowed us to bypass the inhibitory upstream open reading frame of PD-L1 mRNA in the integrated stress response (ISR) activated by impaired haem production, leading to enhanced PD-L1 translation (87). CMTM6 prevents PD-L1 from lysosomal-mediated degradation, which colocalizes with PD-L1 in the plasma membrane and circulating endosomes (88). In addition, Aleksandra developed a pro-code/CRISPR screen that identified socs1 as a negative regulator of PD-L1 (89). In the same way, a study identified IRF2 in a CRISPR-based forwards genetic screen that controlled MHC-I Ag presentation and PD-L1 expression in HeLa cells (90). CRISPR/Cas9 screening found that the transcription regulator MLLT6 is required for effective PD-L1 protein expression and cell surface presentation in cancer cells. MLLT6 loss mitigates the inhibition of CD8 cytotoxic T cell-mediated lysis (91). In addition, CRISPR/Cas9 screening also found that editing Asf1a (69), PRMT1 and RIPK1 (92), MAN2A1 (93), IFNGR2 and JAK1 (94) increased the sensitivity of tumors to anti-PD-1 or PD-L1 treatment. Tumor cells disrupt T-cell-mediated immune monitoring by maintaining high levels of PD-L1, while CRISPR/Cas9 screening found that the PD-L1 transcription and level, translation levels and maintenance of its stability and expression of oncogenic factors, thus exploring the related regulatory mechanisms of PD-L1 to provide a potential biologic therapeutic target for disrupting PD-L1-mediated tumor tolerance therapy.

## CRISPR/Cas9-Associated PD-1/PD-L1 Editing in Different Cancer Species

**Figure 3:**
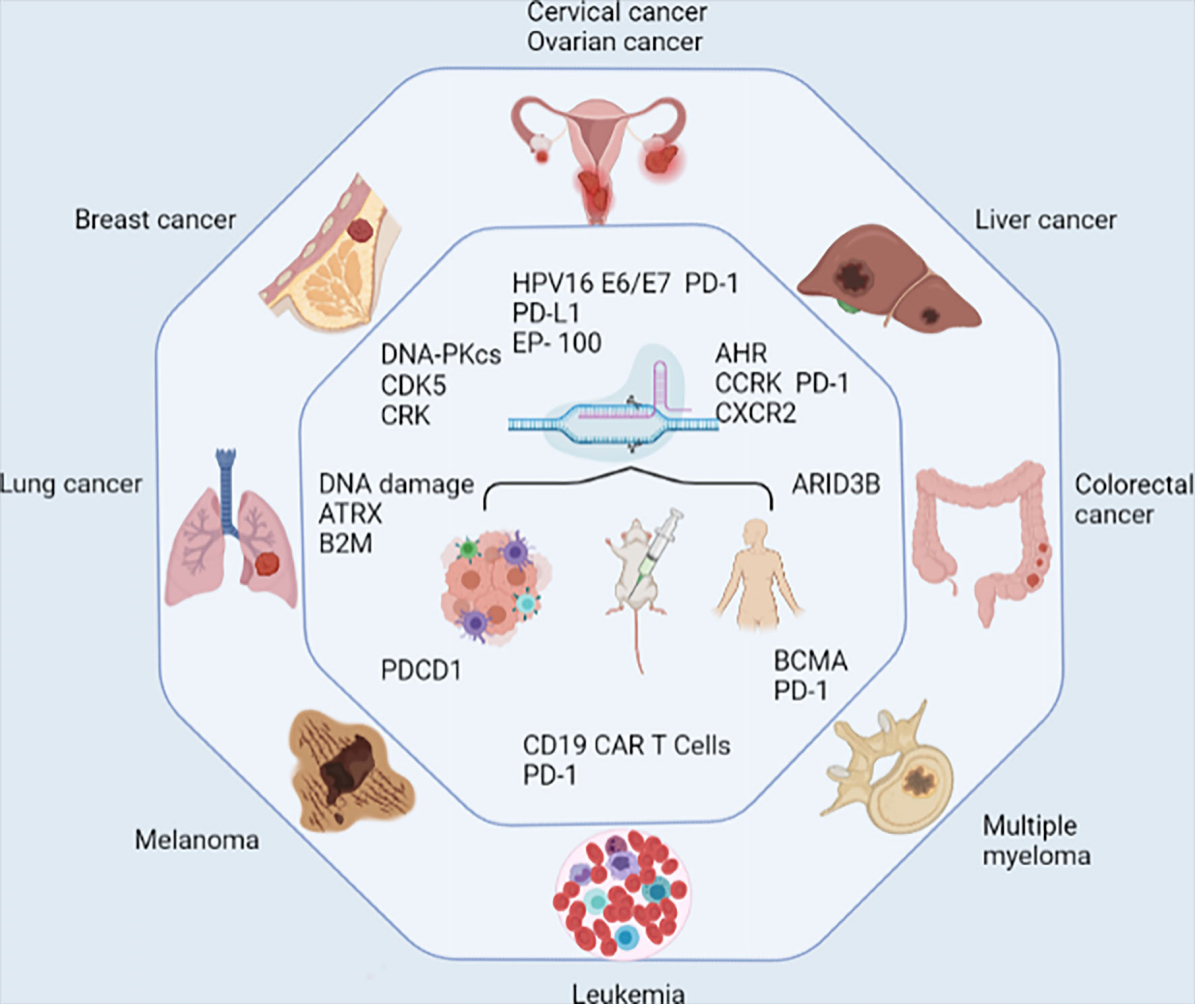
Research progress of CRISPR/Cas9 in different cancer species. CRISPR/Cas9 has been tested at the cellular level, in animals, and even in humans in a variety of cancers, including leukaemia, multiple myeloma, colorectal cancer, liver cancer, cervical cancer, ovarian cancer, breast cancer, lung cancer, and melanoma. CRISPR/Cas9 enhances antitumor immunity by directly editing PD-1/PD-L1 or by knocking out other molecules in coordination with immune checkpoint blockade. Some novel molecules have been proposed as biomarkers for screening people susceptible to anti-PD-1 therapy.

The dominant mechanism of tumor immunity is different in different tumors, which affects the occurrence and progression of tumors in various ways. In this review, we selected several cancers and summarized the role of CRISPR/Cas9 technology-associated PD-1/PD-L1 editing in tumor immunity (**Figure 3**).

### Leukaemia

Leukaemia is a type of hematopoietic stem cell malignant clonal disease with obvious heterogeneity (95, 96). Currently, T cells modified with chimeric antigen receptors targeting CD19 have been developed, which can overcome many limitations of traditional therapies and achieve high remission rates in patients (97, 98). CRISPR/Cas9 technology provides easy access to further optimize the efficiency of CD19 Chimeric antigen receptor T cells (CAR T cells) to meet the needs of routine clinical use (99). The researchers constructed a disposable CRISPR system that easily harvested CD19 CAR T cells and interfered with PD-1 in CAR T cells. In a b-acute lymphoblastic leukaemia xenograft mouse model, these cells significantly increased their antileukaemia activity (100). This suggests that the combination of immune checkpoint disruption may improve the effectiveness of adoptive cell therapy for leukaemia.

### Multiple Myeloma (MM)

MM is a plasma cell malignant proliferative disease with unknown etiology and no cure at present (101). Currently, PD-1/PD-L1 immune checkpoint blockade has shown remarkable efficacy in patients with intractable hematological malignancies (102). Zhao achieved PD-1-deficient CTLs based on CRISPR/Cas9 technology and found that the secretion of TNF-α and IFN-γ increased several times, promoted apoptosis of cocultured MM cells, inhibited tumor growth and prolonged survival within *in vivo* models of MM (54). CRISPR/Cas9 effectively knocked out PD-1 of in CTLs and enhanced their cytotoxicity. This laid the foundation for the potential use of CRISPR in the production of immune checkpoints targeting CAR T cells. However, CAR T cells targeting B cell maturation antigen (BCMA) on the plasma cell surface remain the mainstream treatment plan for MM (98, 103). The safety and efficacy of using CRISPR/Cas9 to edit PD-1 CAR T cells with MM remains to be investigated.

### Breast Cancer

Triple-negative breast cancer is not sensitive to common endocrine therapy and targeted therapy, and resistance to radiotherapy and chemotherapy has become the main obstacle to prolonging the survival of cancer patients, so it is urgent to explore new effective therapeutic targets (104). By using CRISPR/Cas9, Wu found that B7-H1 is related to the catalytic subunit of DNA-dependent protein kinases (DNA-PKCs), which promotes or maintains the activation of ERK or P38 MAPK in cancer cells, thus restoring the sensitivity of cancer cells to chemotherapy (62). In addition, CRISPR/Cas9 knockdown of Cdk5 significantly reduced the expression of PD-L1 on tumor cells, thus effectively inhibiting lung metastasis of triple-negative breast cancer in mice (105). Crk adaptor protein also inhibited EMT and PD-L1 expression on tumor cells through CRISPR/Cas9 gene ablation and inhibited tumor growth and metastasis with anti-PD1 therapy (106). In conclusion, CRISPR/Cas9 compensates for the deficiency that ICIs are basically ineffective in inducing immunogenicity and treating breast cancer and effectively identifies upstream therapeutic targets of immunotherapy, which has considerable potential in exploring and verifying mechanisms of breast cancer metastasis and drug resistance.

### Cervical and Ovarian Cancer

Cervical cancer is the most common gynecological malignant tumor and is highly correlated with Human Papillomavirus (HPV) infection (107). GDNA combined with targeting of PD-1 and HPV16 E6/E7 dramatically increased the number of dendritic cells, CD8+ and CD4+ T lymphocytes and hampered tumor growth (108). Therefore, a reasonable combination of CRISPR/Cas9-mediated PD1 blockade and HPV knockout can have a powerful synergistic effect by increasing the persistence of ICIs on the basis of the high sensitivity of targeted therapy for cervical cancer. In addition, cohort studies have been completed to evaluate the safety and efficacy of CRISPR/Cas9-HPV E6/E7 in the treatment of persistent HPV and HPV-associated cervical intraepithelial neoplasia I (NCT03057912). Ovarian cancer is the main cause of cancer death among gynecological malignant tumors, usually with advanced peritoneal or distal metastasis (109). CRISPR-mediated destruction of PD-L1 on the ovarian cancer cell surface regulates the production of cytokines and chemokines to inhibit the progression of ovarian cancer (58). On the other hand, EP-100 was found to induce PD-L1 expression and immune regulation in LHRH-R positive tumor cells. CRISPR/Cas9 revealed the internal biological mechanism of the synergistic action between anti-EP-100 and anti-PD-L1 by silencing the IL33 gene (110). In summary, CRISPR provides a basis for further clinical research on malignant diseases of the female reproductive system.

### Hepatoma

Aflatoxin is a risk exposure factor for primary hepatocellular carcinoma, and CRISPR/Cas9 genomic screening identified aryl hydrocarbon receptor as essential for hepatocellular carcinoma (HCC) associated Aflatoxin toxicity, suggesting that an anti-PD-L1 immunosuppression regimen can be used as a treatment regimen for Aflatoxin-associated HCC (111). The CRISPR/Cas9 gene editing system disrupted the PD-1 gene in gypican-3 targeted second-generation CAR T cells (112). The role of CCRK and KDM1A in regulating PD-L1 levels on the surface of HCC cells was also explored (113, 114). Moreover, Huang found that Cas9 deletion of PD-1 combined with lentivirus transduction of human telomerase reverse transcriptase can prolong the lifespan of cytokine induced killer (CIK) cells and enhance their antitumor effects (115). In summary, as a more efficient genome editing technology, CRISPR/Cas9 has been widely used in the exploration of immunotherapy for liver cancer.

### Colorectal Cancer

Colorectal cancer (CRC) is a common malignant tumor in the gastrointestinal tract. The occurrence and development of colorectal cancer involves multiple steps, stages and genes (116). Currently, ICIs have been used in the clinical treatment of colorectal cancer, but they are only effective for microsatellite instability MSI patients (117, 118). To explore the precise treatment of ICIs in microsatellite stabilized (MSS) CRCs, Liao discovered the atypical Notch pathway of PD-L1-driven ARID3B, revealing the immune avoidance mechanism of the CRC CMS4 subtype by CRISPR technology (119). These results support the use of ICIs for custom targeting of the CMS4 subtype to screen potential populations that may benefit from ICIs.

### Lung Cancer

Anti-PD-1 immunotherapy has been approved as a first-line treatment for lung cancer patients. However, the response rate was poor in patients with lung squamous cell carcinoma and NSCLC. CRISPR/Cas9 has been explored in many influencing factors. Multiple gene editing of mouse pulmonary organs using the CRISPR/Cas9 system can effectively and rapidly generate LSCC to determine whether induced DNA damage can enhance the immunological characteristics of ICI (64). In addition, CRISPR also validated ATRX as a promising biomarker for ICIs in NSCLC, contributing to patient stratification and decision-making (66). In addition, the inability of antigen processing and presentation caused by damage to the main component of HLA class-I complex B2 M is also explained from the perspective of acquired resistance to anti-PD-1 or PD-L1 antibodies (120).

### Melanoma

Melanoma is a highly malignant tumor derived from melanocytes that is prone to distant metastasis (121). Although ICIs are currently considered the mainstream immunotherapy for melanoma, the response rate of patients to monoclonal antibodies is still not optimistic (120). One study demonstrated the feasibility of CRISPR-edited PDCD1 in effect-memory CTLs with melanoma antigen specificity, and clearly demonstrated its superiority in delaying the growth of PD-L1-positive melanoma (122). Importantly, genes related to metabolism and cell signaling were actively expressed, but genes related to proliferation and DNA replication were downregulated in PD-1-deficient T cell clones. These results suggest that regulation of effector function is more critical than the ability of T cells to proliferate. To further elucidate the effect of metabolic status on the immune response, scientists performed proteomic analysis and CRISPR validation in patients with advanced melanoma who received anti-PD-1 therapy (123). It was finally proven that lipid metabolism, as a regulatory mechanism, increases the immunogenicity of melanoma by enhancing antigen presentation, thereby enhancing sensitivity to T cell-mediated killing *in vivo* and *in vitro*. Therefore, CRISPR can provide insights into the metabolic mechanisms of melanoma immunosuppression to identify therapeutic targets that improve the efficacy of anti-PD-1 immunotherapy.

## Clinical Application of CRISPR Cas9-Related PD-1/PD-L1

**Figure 4:**
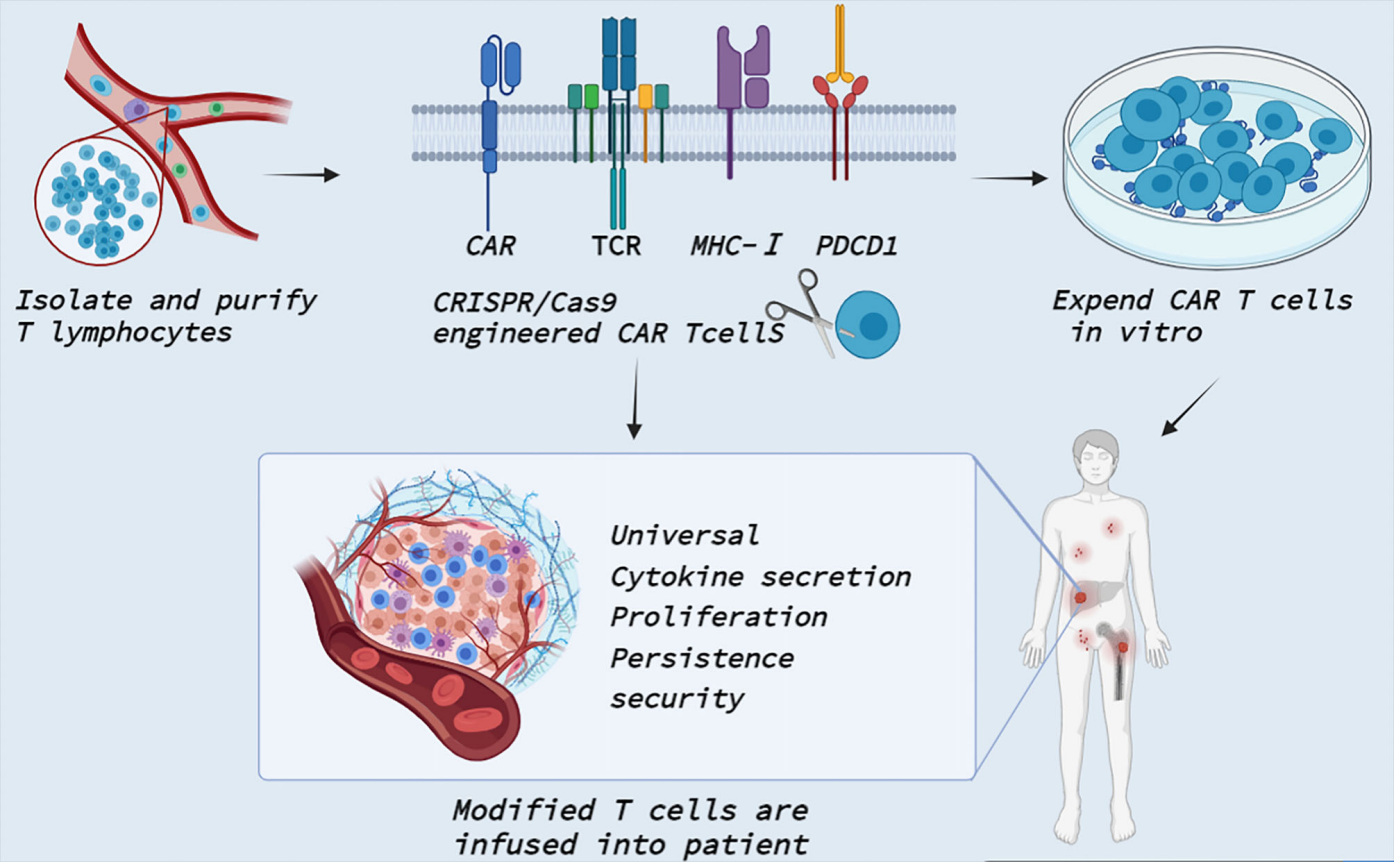
Clinical application of CRISPR Cas9 in CAR T cells. T cells are isolated and purified from healthy persons or patients. CRISPR/Cas9 technology enables precise integration of CAR genes at specific T cell loci. CRISPR/Cas9 also knocked out TCR and MHC-I molecular genes to reduce allogenic reactions and avert GVHD to produce universal CAR T cells. In addition, CRISPR/Cas9 can edit PDCD1 to improve T cell proliferation activity and promote cytokine secretion. The modified T cells are massively amplified *in vitro* and then transfected back into the patient to reverse tumor immunosuppression.

Immunotherapy has become a powerful treatment for many solid and hematological malignancies, including immune checkpoint blocking therapy, immune cell therapy and tumor vaccine therapy (124). Among them, CAR T cell therapy in immune cell therapy has shown great potential in the field of tumor therapy and made unprecedented breakthroughs in clinical practice (98, 125). CAR T therapy is administered by taking T cells from patients and genetically modifying them into chimeric antibody T cells with single-stranded fragment variants from the extracellular antigen recognition domain, the CD3ζ transmembrane domain, and the intracellular T cell activation domain (126). It can specifically recognize tumor cell surface-associated tumor-associated antigen, enhance its defense ability against tumor cells, and stimulate T cell proliferation, cytokine secretion, and lymphocyte recruitment, thereby playing an antitumor role. Fortunately, four generations of evolution have improved the time, difficulty and cost of obtaining CAR T cells, as well as the limitations on the number of patients’ T cells themselves (127). The use of CRISPR/Cas9 gene editing technology opens a new world for the upgrading of fourth-generation CAR T cells (**Figure 4**). Recently, several clinical trials by Stadtmauer (128), Lu (129), Wang (130), and Simon (131) confirmed the clinical feasibility and safety of CRISPR/Cas9- modified T cells.

First, CRISPR/Cas9 can be simultaneously transferred into multiple gDNAs to edit multiple genes at once, providing new ideas for general-purpose allogeneic CAR T cells. For example, Ren by combining CAR lentivirus delivery and electrical transfer of Cas9 mRNA and gRNA, simultaneously targeted endogenous TCR, B2 M and PD-1 and produced allogeneic CAR T cells lacking TCR, HLA class I molecules and PD1 gene destruction. It shows strong antitumor activity *in vitro* and in animal models, with reduced allograft T cell rejection, and does not cause graft-versus-host disease (45). At the same time, they developed a universal system for the generation of CAR T cells by rapid multigene editing, adding multiple gRNAs into CAR lentivirus vectors and redirecting T cells with antigen-specific CAR through a single CRISPR protocol (100). Second, CRISPR/Cas9-mediated disruption of immune checkpoint signaling, such as PD-1, enhances the therapeutic efficacy of CAR T cells in the immunosuppressive tumor microenvironment. Initially, three studies were completed *in vitro* in a preclinical model of human glioblastoma to demonstrate that engineered PD-1-deficient EGFRvIII CAR T cells (132, 133), or CD133-specific CAR T cells (134) showed similar levels of cytokine secretion and improved proliferation and cytotoxicity *in vitro* and enhanced tumor growth inhibition in a mouse model of glioma *in situ*. Similarly, the combination of positive stimulation from CAR and negative regulation of PD-1 by Cas9 blocking can induce T cells to be more persistent and invasive *in vivo* in triple-negative breast cancer TNBC (135). In addition, PD-1-deficient GPC3-CAR T cells significantly increased the phosphorylation of Akt and expression of the antiapoptotic protein Bcl-XL, thus preventing the depletion of PD-1-deficient GPC3-CAR T cells from confronting hepatocellular carcinoma cells expressing natural PD-L1 (112). Nevertheless, altering the proportion of PDCD1-deficient CARS affects T cell metastasis, meaning that a strong loss of PDCD1 may enhance CAR T cells in the short term but ultimately make the edited cells more likely to fail or function impaired. Therefore, further studies on the interaction of tumor load, T cell number and editing frequency in different tumors are needed to ensure that CRISPR/Cas9 plays a positive role (136). Subsequently, the application of Cas9-edited PD-1 in CAR T cells has been progressively implemented in clinical trials such as phase I clinical trials of multiple myeloma with mesothin-positive solid tumors (NCT03545815), multiple myeloma (NCT03399448), esophageal cancer (NCT03081715), metastatic NSCLC (NCT02793856), EBV (Epstein-Barr virus)-positive advanced stage malignancies (NCT03044743), advanced hepatocellular carcinoma (NCT04417764) and engineered TILs/CAR-TILs to treat advanced solid tumors (NCT04842812). Overall, CRISPR technology has brought new vitality to CAR T cells in the field of tumor immunotherapy and has gradually entered the stage of clinical trials.

## Conclusion

In summary, CRISPR/Cas9 gene editing technology has great potential in the field of tumor PD-1-related immunotherapy. CRISPR/Cas9 gene editing technology can directly edit PD-1/PD-L1 or indirectly regulate antigen presentation and upstream and downstream signaling pathways to improve a variety of immune cells and the tumor microenvironment, and combat tumor immunosuppression. CRISPR genome-wide screening technology also provides new ideas for studying upstream and downstream regulatory mechanisms of PD-1/PD-L1, screening synergistic targeting molecules and identifying potentially sensitive patients. CRISPR/Cas9 has clinical value in the diagnosis and treatment of leukaemia, multiple myeloma, breast cancer, cervical cancer, ovarian cancer, liver cancer, colorectal cancer, lung cancer and melanoma. CRISPR/Cas9 produces allogeneic general-purpose CAR T cells combined with immune checkpoint knockout, expanding the clinical application of engineered cells.

However, the following problems need to be overcome when transforming from laboratory to clinical application: First, off-target effects: gene mutations caused by off-target cutting are prone to cellular malignancy but lead to cancer. Therefore, it is necessary to design sgRNA sequences to increase the specificity of target site cleavage (13) and develop Cas9 variants with PAM preference changes (137). Alternatively, the function of the P53 gene should be monitored to prevent toxicity caused by P53 mutations (138). Second, the activity and proliferation of edited cells should ideally be unaffected. Currently, gene editing and cell amplification can be performed *in vitro* and transfused back into patients for treatment combined with adoptive cell therapy. Third, safe and effective *in vivo* delivery methods were selected. At present, the most conventional delivery system is viruses, but they have the disadvantages of limited delivery scale and long-term accumulation easily leading to untargeted cutting. In addition, electroporation and nucleotide transfection techniques have been further explored in animal models. At present, lipid nanoparticle-mediated nonviral delivery without limitation of immunogenicity is more promising (139). Last, Cas9 is an inherent immunogenicity of foreign proteins. Current studies have shown that Cas9 can eliminate immune dominant epitopes through targeted mutations while retaining its function and specificity (140). In brief, although CRISPR/Cas9 gene editing technology shows great promise for PD-1/PD-L1-related therapies in tumor immunity, it is still in its infancy and requires a more comprehensive and in-depth investigation of its safety and reliability.

## Author Contributions

ZS provided direction and guidance throughout the preparation of this manuscript. YX wrote and edited the manuscript. CC and YG reviewed and made significant revisions to the manuscript. SH collected and prepared the related papers. All authors read and approved the final manuscript.

## Funding

This study was supported by The National Natural Science Foundation of China (81972663, 82173055, U2004112), The Excellent Youth Science Project of Henan Natural Science Foundation (212300410074), The Key Scientific Research Project of Henan Higher Education Institutions (20A310024), The Youth Talent Innovation Team Support Program of Zhengzhou University (32320290) and The Provincial and Ministry co-constructed key projects of Henan medical science and technology (SBGJ202102134).

## Conflict of Interest

The authors declare that the research was conducted in the absence of any commercial or financial relationships that could be construed as a potential conflict of interest.

## Publisher’s Note

All claims expressed in this article are solely those of the authors and do not necessarily represent those of their affiliated organizations, or those of the publisher, the editors and the reviewers. Any product that may be evaluated in this article, or claim that may be made by its manufacturer, is not guaranteed or endorsed by the publisher.

## Abbreviations

CRISPR/Cas9, Clustered regularly interspaced short palindromic repeats/CRISPR-associated nuclease9; PD-1, Programmed cell death 1; PD-L1, Programmed death ligand 1; CTLA4, Cytotoxic T lymphocyte protein 4; sgRNA, Single-stranded guide RNA; PAM, Protospacer adjacent motif; DSB, Double-strand break; NHEJ, Non-homologous end-joining; HDR, Homology-directed repair; TALEN, Transcription activator-like effector nuclease; TIL, tumor-infiltrating lymphocytes; NSCLC, non-small cell lung cancer; DC, dendritic cells; CTL, Cytotoxic T lymphocyte; ADCC, Antibody-dependent cell-mediated cytotoxicity; ICI, Immune checkpoint inhibitor; LSCC, Laryngeal squamous cell carcinomas; HNSCC, Head and neck squamous cell carcinoma; TAM, tumor-associated macrophages; NAMPT, nicotinamide phosphoribotransferase; ISR, integrated stress response; CAR T cell, Chimeric antigen receptor T cell; BCMA, B cell maturation antigen; DNA-PKC, DNA-dependent protein kinase; HPV, Human Papillomavirus; HCC, hepatocellular carcinoma; CIK, cytokine induced killer; CRC, Colorectal cancer; MSI, microsatellite instability; MSS, microsatellite stabilized.
